# What Makes Integration of Chronic Care so Difficult? A Macro-Level Analysis of Barriers and Facilitators in Belgium

**DOI:** 10.5334/ijic.5671

**Published:** 2021-10-29

**Authors:** Katrien Danhieux, Monika Martens, Elien Colman, Edwin Wouters, Roy Remmen, Josefien van Olmen, Sibyl Anthierens

**Affiliations:** 1 University of Antwerp, Family medicine and Population Health, Doornstraat 331, 2610 Wilrijk, BE; 2 Institute of Tropical Medicine, Department of Public Health, Kronenburgstraat 43, 2000 Antwerpen, BE; 3 University of Antwerp, Centre for Population, Family & Health, Prinsstraat 13, 2000 Antwerpen, BE

**Keywords:** integrated care, health care policy, chronic care, stakeholder interviews, governance, health care systems

## Abstract

**Introduction:**

Although many countries have been implementing integrated care, the scale-up remains difficult. Macro-level system barriers play an important role. By selecting three key policies, which have implemented integrated care in Belgium over the last 10 years, we aim to go beyond the identification of their specific barriers and facilitators to obtain an overarching generic view.

**Methods:**

27 participants were purposefully selected, to include all important stakeholders involved on the macro-level in chronic care in Belgium. Semi-structured interviews were guided by a timeline of policies and an inductive thematic analysis was performed.

**Results:**

Barriers and facilitators were identified on both health care and policy level. The major factors restraining the scale-up of integrated care are the fee-for-service reimbursement system, limited data sharing and the fragmentation of responsibilities between different levels of government. Remarkably, these factors strongly interact.

**Discussion:**

This paper highlights the importance of homogenization of responsibilities of governments regarding integrated care and the interdependency of policy and health care system factors. A whole system change is needed instead of the current Belgian model of prolonged search for common ground between conflicting opinions. Political commitment and citizen participation will be crucial.

## Introduction

Throughout the world, health care systems are struggling with an ageing population, increasingly required to care for patients with multiple chronic diseases. Both the long-term aspects of these diseases and the complexities that arise when they coincide put health care workers under additional strain, especially when working in a system not designed to meet these new challenges. Over recent years, integrated care has gained attention as a potential answer to these challenges [1]. Integrated care involves health services that are managed and delivered in a manner that provides people with a continuum of health promotion, disease prevention, diagnosis, treatment, disease management, rehabilitation and palliative care services, coordinated across different levels and sites of care, within and beyond the health sector, and according to individual needs throughout the life course [2]. The key aim is to overcome the current fragmentation of care, which is often too episodic and provider oriented.

In recent decades, numerous integrated care projects have been rolled out and researched [3,4,5]. Models of integrated care have proven to be able to improve patient satisfaction, perceived quality of care, and to enable access to services [6]. Despite global consensus on the need for integrated care [7], implementation and scale-up in many countries is constrained by various barriers and challenges [3,8]. Such barriers are linked to gaps in leadership, organizational culture, information technology, communication, capacity, resources and provider commitment [9,10,11,12,13]. The attempt to define the significant elements of integrated care models has not notably led to more successful scale-up, changing the focus to the context in which they should operate. Moreover, there is a gap in the literature on these macro-level factors, such as legislation and policies to support integrated care or its financing [14]. Therefore, both Struijs et al. [15] and Minkman [16] have urged for more research to be undertaken on macro-level contextual factors such as governance and payment models.

Currently, the research has shifted its attention to this macro-level context, exploring effect differences of similar integrated care models in cross-country projects [17,18,19,20]. However, these studies have various limitations. Firstly, findings are often confined to one particular model, or focus on one policy, identifying the barriers and facilitators in that particular project. This might not be sufficient, as such studies ignore the complex reality in most countries, where many different projects and policies are proceeding concurrently and interact with each other. As projects do not take place in a static controlled context and the concurrent introduction of new policies might have implications over time, the effect of macro-level factors on these projects might also change over time. Additionally, such well-defined projects often focus on a distinct but limited group of beneficiaries, while in a subsequent phase of scale-up to the entire population, the effects and barriers differ in size and nature.

This paper answers the need for a broader scoped macro-level analysis of integrated care implementation. It provides a comprehensive overview of the barriers to and facilitators of a scale-up of integrated care in Belgium viewed from the macro-level context. We aim to analyse the most prominent integrated care policies in the field of health policy on chronic care in Belgium and to assess the barriers to and facilitators of policy development and implementation from the perspective of key stakeholders. By selecting three key policies in the field of integrated care from the last 10 years, we aim to go beyond the identification of the specific barriers and facilitators in each case to obtain an overarching generic view. The goal is to build our understanding of why health care systems have not yet made a real shift towards integrated care, from the perspective of macro level stakeholders.

## Background

Belgium is a federal state with a federal government and three territorially based regions (Flanders, Wallonia and Brussels-Capital), as well as three language-based communities (the Flemish, French and German communities), as a whole referred to as federated entities. This complex governing system has resulted in nine ministers of health for a country of 11.5 million inhabitants. The Sixth State Reform, which came into force in 2014, reallocated some responsibilities from the federal level to the communities, which are now responsible for long-term care, elderly care, primary care organization, disease prevention and health promotion. The federal government remains responsible for curative care, including the payment of primary care providers and hospital care, which makes up the bulk of the health care budget.

Health insurance in Belgium is compulsory and managed by the National Institute for Health and Disability Insurance (NIHDI); every Belgian citizen must register through one of the six national sickness funds. Providers are largely paid through fee-for-service payments and have a large degree of therapeutic freedom. People have unrestricted access to any health care provider at all levels. However, patients have a relatively high out-of-pocket expenditure compared to other European countries, as this accounts for 18% of total health care expenditure [21]. Decision-making about financing relies on negotiations between several stakeholders [22]. As such, health insurance and its budget are decided through national conventions and agreements bilaterally by the sickness funds and by various ‘syndicates’ or associations representing health care professionals, employers, salaried employees and self-employed workers. Afterwards, the federal Minister of Health decides to accept these conventions or not.

Despite the health status of the Belgian population generally being good, the OECD health care system analysis pointed to several weaknesses, such as low expenditure on prevention [21] and an important share of citizens delaying contact for financial reasons [23]. Mortality from treatable causes is low, but preventable mortality and avoidable hospital admissions are higher than in many other Western European countries [21]. This indicates that the health care system may effectively address acute health problems, but that there is a performance gap with respect to other countries in reducing premature deaths linked to chronic diseases.

**Figure 1:**
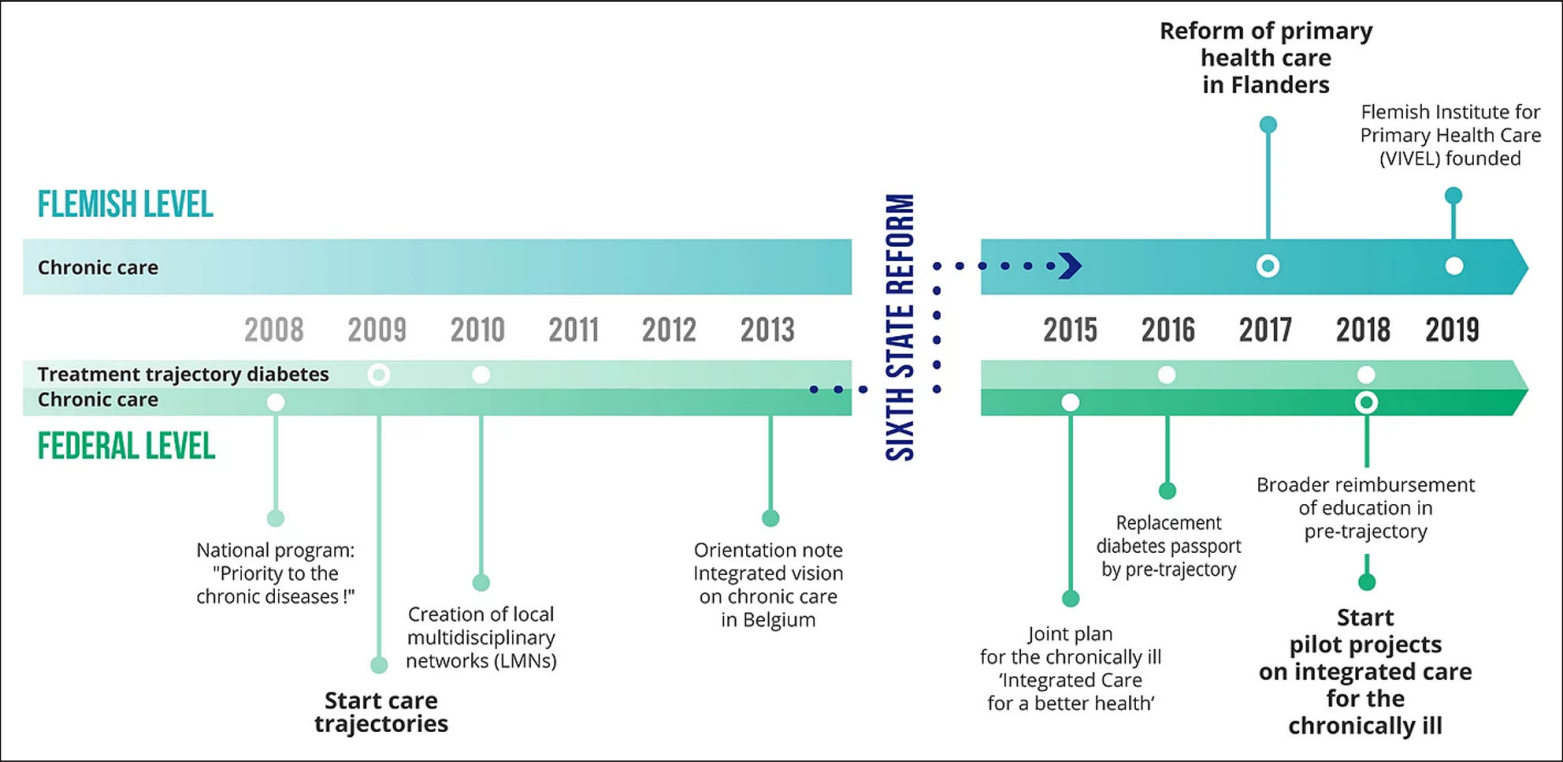
These three key policy initiatives guided our stakeholder interviews.

In response to these weaknesses, integrated care has been put on the policy agenda, for more than a decade. The most important policy initiatives are summarized in the timeline in ***Figure 1***. The first step towards integrated care was the *Care Trajectory*, which was introduced by the NIHDI for type 2 diabetes and for kidney failure in 2009. It was based on the Chronic Care Model [24] but was disease-specific and only included a limited subset of patients in the advanced stage of each disease. As part of this policy, a contract between patient, GP and specialist is used to support interaction between these three actors [25]. Financial incentives are provided for the physicians and better access to self-monitoring material and education is granted to the patient. Local multidisciplinary networks support the implementation of the care trajectory.

Meanwhile, the federal Minister of Public Health decided to give priority to chronic diseases and requested in 2010 that the Belgian Health Care Knowledge Centre drafted a vision for the future for chronic care. The Centre synthesized national and international research and also explored the views of stakeholders, which resulted in a position paper with more than 50 recommendations tailored to the Belgian situation [13]. The rising prevalence of chronic diseases and the budgetary impact impelled the federal and regional governments to develop a common orientation policy note on chronic care, which was fed by the position paper. The field of prevention was added to the scope and resulted in 20 concrete actions to be taken. After the Sixth State Reform, the new ministries of health of the different governments agreed on a joint plan for the chronically ill, which took into account the ‘Triple Aim’ [26]. Its vision was that an integrated approach in medical, paramedical, psychosocial, nursing and social care is needed to guarantee coordinated service provision, including a new way of financing chronic care [27]. Part of this plan involved the *Pilot projects in integrated care* [28], which have the aim of testing this new approach and started in 2018. In this regard, consortia consisting of local actors could submit a project, of which 12 were chosen to be implemented.

Concurrently, the Flemish government decided on its *Reform of Primary Care in Flanders* in 2017 [46,47], aiming at more integrated and person-oriented care, based on patient need rather than supply capacity. The reform consists of different projects, with the development of primary care zones as central component. These new structures each serve a population of 75,000 to 125,000 inhabitants and have the task of supporting multidisciplinary and intersectoral collaboration. They are to be directed by a ‘Care Council’, including representatives from local authorities, the health sector, the social sector and people in need of care and support.

## Methodology

### Design

A qualitative research design is the most appropriate to study the ideas and opinions of stakeholders. As the aim was to unravel the perceptions and difference in perceptions of individuals in particular, interviews were the most appropriate method. This study is part of two research projects: SCale-Up diaBetes and hYpertension care (SCUBY) project [29], which aims to scale-up integrated care through the development and evaluation of roadmap strategies in different types of health care systems in Belgium, Cambodia and Slovenia; and the federated consortium for appraisal of integrated care teams in health in Belgium (FAITH.be) [28], whose aim is to evaluate the pilot projects on integrated care.

### Stakeholder selection

The selection of stakeholders was performed in a cyclical process, guided by the WHO Stakeholder analysis guidelines [30]. Firstly, a list of all possible stakeholders was developed. Secondly, with the input of two experts acquainted with the players in the field of Belgian health care policy, this list was narrowed down to 22 priority stakeholders, focussing on those with most power and interest in chronic care. As this field is extensive and complex, a choice was made to focus on mainly Flemish and Dutch-speaking stakeholders. Thirdly, snowballing was used as a method through which interviewees could refer us to other key stakeholders, which led to four more participants being added. Stakeholders were contacted by e-mail or phone. All stakeholders accepted the invitation or provided a substitute within their organization. One stakeholder retracted participation after the interview; two participants included another colleague in the interview. Therefore, 25 interviews of 27 participants were used in the analysis. The final list of organizations with participating stakeholders can be found in ***Table 1***.

**Table 1:** Organizations and participating stakeholders.


ORGANIZATIONS	

**Regulatory stakeholders (policymakers & public administrators)**	

Federal Public Service of Health (FOD)	Flemish Cabinet

Association of Flemish Cities and Municipalities (VVSG)	Flemish Agency of Care and Health (VAZG)

**Finance stakeholders**	

National Institute of Health & Disability Insurance (NIHDI) (3 interviews)	Christian Health Fund (CM)

Joint College of Sickness Funds (NIC)	Socialist Sickness Fund

**Provider organizations (labour – professional associations)**	

Medical Association of GPs (Domus Medica, DM)	Flemish Association of Dieticians

Belgian Association of Doctors Syndicates (BVAS)	Association of Diabetes Nurses

Medical Association of GPs and Specialists (ASGB)	Flemish Association of Independent Nurses (VBZV)

General Pharmaceutical Association (APB)	Network of Homecare Nurses (Zorggezind)

Network of Hospitals (ICURO)	Association of Home Nursing (WGK)

**User and patient groups**	

Flemish Patient Platform (VPP)	Flemish Diabetes Association (Diabetes liga)

**Scientific stakeholders**	

Federal Knowledge Centre for Health Care (KCE)	Academia/Medical universities (2 interviews)


### Data collection

Semi-structured face-to-face interviews were carried out by the two first authors, KD (general practitioner) and MM (public health scientist), from April to September 2019, at the stakeholders’ offices or at a place convenient for them. The interviews lasted 66 minutes on average and were audio-recorded and transcribed verbatim. The main topics of the interview guide were: stakeholder role, their understanding of integrated chronic care, and the barriers to and facilitators of the development and implementation of integrated care policies. We used a visual timeline (***Figure 1***) to introduce the three main policies on integrated care to guide the interview. This approach helped to clarify the policy context for the stakeholders interviewed and facilitated the discussion of their perceptions of these integrated care policies and existing systemic barriers. Furthermore, stakeholders were also asked to score the current state of Belgium’s implementation of integrated care on a scale from 1 to 10. An iterative approach was used, adapting the interview guide slightly, based on the first analyses.

### Data analysis

An inductive thematic analysis [31] was performed to identify barriers to and facilitators of the implementation of integrated care. All of the interviews were read by the first two authors (KD and MM) to immerse themselves in the data, after which initial codes were formed and agreed upon in the wider interdisciplinary team, with each team member reading through an allocated number of the interviews. Subsequently, the dataset was coded in a systematic fashion, with the codes representing the different barriers to and facilitators of the scale-up of integrated care. New codes arose during this process and all were grouped into a thematic framework, with main and sub-codes. Researcher triangulation was carried out through discussion of the data at several stages in the analysis process by the wider multidisciplinary team, comprised of general practitioners, sociologists and a public health scientist. A codebook that defined each code and subcode was developed. The final code tree can be found in ***Appendix 1***. The codes were then used to create a scheme, explaining their relationships. This scheme was repeatedly discussed in the wider team until consensus was reached. Finally, the interviews were reread to check whether the final scheme was grounded in the data. NVivo version 12 was used to support the analysis process. An additional stakeholder analysis was conducted on the position, interest and power of the different stakeholders [48].

The Ethics Committee of Antwerp University Hospital approved the study (registration number B300201940005), and the interviewees provided informed consent.

## Results

The barriers and facilitators identified in the interviews were classified into three kinds of factors: 1) integrated care factors; 2) health care system factors, consisting of barriers linked to health care delivery, data sharing and the health care payment system; and 3) policy factors, including fragmentation of responsibilities, participation and political culture. The following sections highlight the variability in stakeholders’ views on these factors.

### Integrated care, the ultimate goal

Stakeholders had diverse interpretations of integrated care, which was also recognized by some stakeholders as an implementation barrier. To describe integrated care, interview participants used various terms, including: comprehensiveness, continuity, cooperation, accessibility and patient-centredness. Despite their use of similar and related concepts, the extent or depth of integrated care implementation that they envisioned differed. Some stakeholders expressed they were satisfied with a project promoting interprofessional cooperation, while others believed integrated care should go as far as transforming the entire health care system.

*“Some people only see it as tackling a transmural project and that’s where it stops.” (IV10)*

Additionally, as one participant reflected, since there are so many different objectives, it would be impossible to attain them all at the same time.

*“You cannot reach the same objectives of integrated care, continuity, comprehensiveness, person-centred, community-centred, it’s impossible to reach these different outcomes or goals at the same levels. So, when you speak about integrated care, you must choose a theory which is the package of activities you will include in it.” (IV26)*

Despite these variations in perceptions on integrated care, stakeholders largely agreed that implementation of integrated care in Belgium is suboptimal: most stakeholders gave a low score to the model’s current implementation in Belgium (median = 3.5/10, IQR = 3–5/10).

### Health care system factors

#### Health care payment system

The financing system was most often indicated as the main factor affecting integrated care. The stakeholders argued that money is a prime incentive determining the behaviour of health care workers. Most participants agreed that the current provider payment mechanism system hinders care from becoming integrated because, as a predominantly fee-for-service system, it incentivizes each individual service. The system is grounded in acute care but has not been adapted to the current epidemiological context, where care for chronic patients is becoming a major domain of primary care providers. In addition to medical consultations with the patient, chronic care requires multiple other tasks, such as follow-up and coordination. These tasks are currently not reimbursed. Furthermore, a fee-for-service model does not stimulate cooperation and referral, since referring a patient to somebody else means potentially cutting the health worker’s own income.

*“I think the financing model is really one of the triggers to change something. The fact is that every health care provider performs a procedure and is financed solo for it. Whereas I think you really should go to a financing model that also stimulates and rewards cooperation and referral. One that also rewards you for following up a patient as a whole, not for the specific moment when they come for the care they need, but for the whole follow-up.” (IV14)*

To overcome this challenge and to move beyond the traditional provider payment method, most participants argued for a combination of remuneration on a fee-for-service and a capitation basis. Maintaining a mixed system was considered important, as it was feared that a full capitation-based system would affect the commitment and work attitude of health care workers.

*“In other health care systems, where doctors are much more financed within a capitation system, the door closes at 6 p.m., and there are drawbacks to this too. So, I think that this needs to be looked at in a balanced way, but it is indeed the case that, when it comes to integrated care, one should be creative to find good solutions within that system to make integrated care possible. In that respect, I think it is positive that, in these pilot projects, people have also consciously given room to experiment with financing models.” (IV16)*

One proposal made by some stakeholders was to pilot this new hybrid provider-payment model in a small population, such as patients in need of chronic care. Other refinements to the payment system to facilitate integrated care across organizational boundaries were mentioned, such as funding teams or interorganizational collaborations rather than individual providers, as well as incentivizing the quality of care. However, the lack of agreement on measurable and meaningful quality indicators remains a hurdle to determining and thus rewarding the quality of integrated care. This mixed provider payment method is being tested in pilot projects in integrated care.

*“I think you have to look at it for a delineated group of people diagnosed with a chronic illness. You don’t have to do everything per procedure for this group. And I am not in favour of the complete abolition of [task] performance medicine, not at all. But, if you just look hypothetically at that group involved in primary care, to give a bit of a lump sum. Because you know that for these people you need a bit of extra consultation with other health care workers, extra communication anyway, which is something that health care providers complain about: this is not part of the procedure that I actually charge for.” (IV8)*

Finally, some of the participants mentioned an area of tension between health care workers who work as employees and those who are self-employed. The implications of any system change will differ for health care workers depending on their employment status, and therefore their stake in reforms is different. Cooperation between the two different groups may be difficult as the employees prefer to hold meetings during working hours, whereas self-employed practitioners prefer out-of-hours meetings. If self-employed practitioners are not paid to attend and voluntary commitments are required for any move towards integrated care, their motivation may decrease.

*“That’s what you have with the self-employed. It’s hard to get in for free.” (IV1)*

Thus, this tension between groups with different employment status is problematic, as everyone needs to be on board to move towards integrated care.

#### Health care delivery

Three themes emerged from the analysis of this factor: 1) the organization of care based on health needs; 2) the balance in curative and preventive care; and 3) task substitution.

Firstly, many stakeholders commented that clarity about the needs, activities and responsibilities in delivering an integrated care package would be crucial to make progress in any reorganization. This has been done in the disease-specific diabetes care pathway, but integrated care has a wider scope. Respondents noted the necessity of moving beyond a supply-based approach to single diseases towards a holistic needs-based approach. This implies that health care providers gain a better insight into the needs of their patients, or – even more far-reaching – into the needs of the population in their area or under their responsibility. Currently, the focus of health care professionals is on caring for the patients coming to their practice. Therefore, according to our participants, to consider these needs, a strategy such as ‘population health management’ would be innovative in Belgium and extremely important for health care professionals to become acquainted with. To facilitate this population-based approach, implementers need to understand these needs and know what is available on the supply side of integrated care. In this regard, while some minimal data has been collected to date, this is not available to individual health care workers or to the local regions. Stakeholders mentioned that the new platform designed for primary care zones is a promising opportunity to introduce a population-based approach in Flanders.

*“A second important pillar is the development of operation-oriented care and the associated data access. In our agency, the department of information and care professions are working very hard to bundle data, to aggregate data, to scroll through different data and to make them available to the individual care councils and the care actors via the Flemish Institute. I think that unlocking data can offer an enormous opportunity to stimulate quality thinking both at the level of primary care zones and at the level of individual practices.” (IV24)*

Secondly, many participants stated that the visualization of needs from a population perspective would shift attention from curative health care services to needs in the field of health prevention and promotion. This implies a shift in resources from secondary care to primary care and a rethinking of the division of tasks between different professionals working in the health system.

*“You know that if you move towards integrated care, which is needed now, then a shift from secondary to primary care will be needed.” (IV11)*

Thirdly, many stakeholders argued that task shifting is essential in order to adapt to current patient/population needs. Currently, in Belgium, numerous tasks are performed by health care providers who are overqualified. However, the efficient division of tasks is blocked by the law on the execution of tasks by health care providers as well as by reimbursement rules, as some procedures are only remunerated when performed by a physician.

*“They now have implemented the recognition of specialist nurses, giving them other tasks. But if you then see that those specialized nurses would be absolutely welcome within that first line but in fact we can’t pay them, because there is no nomenclature [i.e. regulation on medical professional task allocation] for this, […] in this way you have a problem.” (IV18)*

#### Data sharing

A recurrent theme was the need for a health information system allowing the sharing of data between providers and with the patient. A well-functioning digital system, which allows linkage between different types of providers was considered an important prerequisite for integrated care. Marketization has led to many different digital solutions developed by a variety of commercial players which provide different software packages for primary care professionals. Many of these packages cannot link or exchange data with other relevant systems. This is the case within and between professional groups in the medical sector, and an even greater issue in relation to coordination with the input of the patient and the social sector, as these actors are completely disconnected from the platforms used to share information. The collaboration with the social sector especially has thus far received very little attention, despite integrated care implying collaboration between care providers and the patient in their home environment, which often requires interaction with home-based caregivers and social workers.

While a digital health strategy has been formulated and implemented by the federal and regional governments, the respondents reported uncertainty about the elements of implementation. Privacy issues and legislation remain unclear and at political level as well as among stakeholders, there is disagreement about whether to pursue a common, centrally governed system for the entire health care system or to further stimulate the role of private companies competing on the free market. Proponents of a centrally governed system argued that the Belgian market is too small for multiple developers to sell their packages and be qualitative and innovative, especially when having to comply with complex regulations.

*“The same counts for a lot of the health care providers, who should be able to access certain information because they need it to provide the highest quality of care. But currently it is not allowed by privacy laws. And incomprehensibly perhaps.” (IV25)*

*“What I find unfortunate is that one of the 14 components [of the common plan for integrated care] is the electronic patient record, or the integrated patient record. The government’s ambition was to offer this. Internally, we had said we were going to do it, but externally mainly the cabinet did not; the projects had to do it themselves, the market had to play.” (IV17)*

### Policy factors

#### Fragmentation of responsibilities

The current state structure of Belgium – which implicates that different governments are responsible for different aspects of the care continuum – was identified by numerous participants as a key barrier to integrated care. While the Federal and Flemish governments have shown and expressed their commitment to implementing integrated care, they face the fact that they cannot control an important part of the care continuum, as responsibilities are fragmented across different levels and there is no one structure that can make decisions concerning the whole system. Taking this into account, as well as the fact that the whole care continuum is a key aspect of integrated care, governments will thus have to cooperate in the current structure, which remains difficult, according to the participants.

*“How can you find good solutions if you have nine ministers of health for 11.4 million Belgians? I always compare it with China, it’s an easy rule of three. There are 1.43 billion Chinese. […] So, you would need 1,100 ministers of public health in China.” (IV7)*

*“It’s very fragmented of course. It’s almost nonsense to implement an integrated care project in a country like Belgium. Particularly if you want to put in your package […], if you want to put health promotion activities, health prevention activities and curative activities, which is what integrated care projects want to do. It’s just impossible.” (IV26)*

The distribution of responsibilities also leads to confusion, as the system is too complex, which creates frustration. One example of the complexity is apparent in the fact that at both the federal and the Flemish levels territory is divided into different areas within which care should be organized. However, these areas each have different demarcations, for example the Primary Care Zones created as a part of the Primary Care Reform do not overlap geographically with the loco-regional areas in which the pilot projects which flowed out of the National plan operate. This hinders cooperation on a local-regional level and slows down improvements.

*“Another observation is that, even on the management level of large organizations, people are insufficiently aware of the regulations in Flanders, and more than likely also federally, of what is possible and what is not. People often assume things are probably not possible whereas they are.” (IV11)*

*“The Flemish primary care zones do not correspond to the hospital networks. Everything is possible, but it does make things more complicated. It will work out, but it will be more complicated than if it had not.” (IV16)*

In addition, the separation of preventive and curative domains across different governmental levels has implications for resource allocation. Investment in prevention or health promotion are expected to lead to cost-savings in the curative domain in the long run. However, these investments are borne by the regional governments, while cost-savings will be made at the federal level. Consequently, the Flemish government is not stimulated to invest money on prevention if they do not receive any financial benefits.

*“The greatest efficiency would be putting the entire money pot together and daring or being able to accept that you might have an increased cost in one part, but it will be lowered somewhere else. And of course this is also politically difficult. I think this is one of the frustrations of Minister Vandeurzen [Flemish Minister of Health, 2009–2019], who has put a lot of effort into prevention. I can’t read his mind and I can’t speak for him either, but the profits you gain through prevention are federal because that’s where the expenditure still is and the benefits are.” (IV6)*

Most participants felt rather hopeless about the effect of the fragmented state structure, as they could not see an easy solution to this problem. The current strategy to overcome these barriers is concertation via the Interministerial Conference, in which the different ministers of health consult and negotiate with each other. However, participants considered this inefficient. Therefore, most stakeholders suggested that all responsibilities should be assembled at the same policymaking level. The specific level of homogenisation of responsibilities was of minor importance to our respondents.

*“Two solutions: everything on federal level or everything on regional level and all models in between are doomed to disappear.” (IV14)*

#### Participation

The respondents stated that the participation of different stakeholders from the field in policymaking is paramount, but that on the federal level the current model of the NIHDI, in which concertation and consensus are central, is paralysing the system. Firstly, there are too many boards and councils, which slows down the decision-making process. Secondly, budget allocation within the NIHDI is fragmented and therefore problematic. The global budget is first allocated to the different professional groups, who subsequently decide on how it will be spent within their own medical profession. Both aspects – the number of decision-making organs and the (siloed) budget allocation – hamper cooperation and integrated care. In addition, the lack of representation of patients and of political representatives within the NIHDI were mentioned as problematic.

*“You’re going to have to reform the whole concertation model anyway. I am very much in favour of social consultation between care providers, sickness funds and the government. But the silo model within the NIHDI model, where you start a discussion with a certain type of health care provider about how you are going to distribute the money. First of all, when you discuss diabetes within the Medicomut [insurance committee at NIHDI consisting of physicians and sickness funds], you overlook essential partners. You also need the podiatrists, you need the dieticians, so you can make great plans, but if those caregivers in their convention say we won’t do anything with it, then you just come to a stand there. So, I think in that concertation model you’re going to have to take out the silos and put in some partners. Who’s completely missing there, the patients themselves. They’re completely missing. And secondly, and that’s not something people like to hear so much, but I think we’ve evolved in such a way that you actually have to give politics a place in such a concertation. So that you have to give your administrations and your cabinet a place, because there’s no point today in discussing it among yourselves and then always getting mixed up, politically, financially, budget wise.” (IV2)*

At the Flemish level, representation is organized differently. Since 2019, as part of the Flemish primary care reform, care councils have been set up locally, according to the principle of participation: they bring together representatives of health care workers, social workers, patients and the local government.

All stakeholders mentioned a preparedness for change that is needed to implement integrated care among health care workers in their practices. At present, integrated care is a hot topic in the field, and according to most participants the sense of urgency is rising. Health care workers want the best for the patients they care for and the interviewees from professional associations mentioned that in their experience the current model is not optimal to achieve this goal.

*“I think most doctors, nurses and so on are open to positive changes. In the sense of working together. And in the end, I think we’re all still a bit of an idealist, better care for the patient. So if there are things that are achievable, that are clear, that are logical, etc., then I think there is still a lot of goodwill present.” (IV4)*

At the same time, stakeholders indicated that health care providers do not want to lose out in any reform and that certain professions fear to lose power, resources, roles or status, while others could gain from certain changes. This area of tension due to corporatism a.o. might block change completely, especially as the professions which have the most to lose have greater power, insofar as they are more strongly represented in decision-making structures.

*“A lot of general practitioners still have problems with multidisciplinary collaboration. So they still have a hard time delegating tasks or the right care in the right place by the right person, which is still difficult for a number of people. […] And if you then have to compete against a syndicate that only wants everything per performance and tries to turn every proposal in that direction.” (IV10)*

#### Political culture

The last theme is political culture, which refers to the ways policy processes evolve, the way stakeholders interact and use power, and to the specific norms and traditions of decision-making in Belgium.

At the health policy level, there is a tendency to develop visions that emphasize values, such as quality, solidarity and empowerment. Position statements coming from different directions, such as hospitals or the academic sector, are being developed and shared. In addition, on the decision-making level, there has been much debate about integrated care for chronic diseases which has resulted in a number of policy plans. However, the respondents indicated their disappointment that so far, the various policy plans and initiatives have not yet resulted in a shared long-term vision, due to the limited whole systems thinking at political level, beyond the borders of a governmental level’s competences and beyond the focus on the next elections.

*“There are holes in the vision story. Yeah? Okay, so now there’s a little bit of a lack of global vision in policymaking. […] Actually, the government should take a little distance first, take a little distance and look again from a global policy vision.” (IV23)*

Moreover, participants complained about the limited resources and time allocated to the support for implementation and evaluation of the policies.

*“But everything always clashes with means, doesn’t it? If you say: ‘We want to do something’, you could say: ‘You know what, we’re going to look at what kind of cooking pots we need, what kind of ingredients we need’. And once you know … You have to say in advance: ‘And we’re going to provide money to buy enough cooking pots and resources’. But most of the time, that is lacking.” (IV22)*

The lack of focus on evaluation also obstructs the improvement or abandonment of mediocre or poorly functioning projects and thus the further pursuit of integrated care.

*“As always. It hasn’t been evaluated. That was the goal, but the first time they got data, they could just throw it in the trash bin. They were just bad. There was nothing you could do with it. […] So yes, the necessary follow-up, adjustment or possibly saying no this project doesn’t work and so stop, that doesn’t happen. […] Had they done more evaluation and adjustment in those ten years, then we would have gotten further than we are today.” (IV2)*

Many respondents referred to the long duration of negotiation processes and the incremental nature of policymaking in Belgium, which is related to the overabundance of governance structures, as described above. This precludes major reform and the investment necessary for a whole system change, which is further hampered by the current budgetary context of austerity.

Furthermore, the current distribution of responsibilities over the federal and regional governments has led to a power struggle between the policy stakeholders at federal and federated levels, as some interviewees described. Although political interview respondents indicated that federal and federated entities are on the same page and jointly striving towards the goal of integrated care, different entities often set their own priorities and primarily want to achieve the best outcomes for their policy for self-serving reasons, to appear successful. Consequently, projects coordinated by another governmental level or entity will not always receive the support needed, as is the case with the pilot projects for integrated care, which currently lack support from the Flemish government.

*“Well, she feels primary care zones are the way to go. And she’s very proud about that of course. But if Integreo [the implementation of the joint/national plan, which produced the pilot projects on integrated care] is going to be a success, which is somehow connected with the primary care zones, it’s not good for her because it’s not the primary care zones, it’s Integreo. […] It’s important to some people to attribute success or failure. Success in what they are doing, failure in what others are doing.” (IV26)*

To address the systemic barriers to change, substantial rules and regulations need to be revised by politicians. This demands decisions on multiple levels and potential resistance at all levels – and thus a considerable amount of political commitment and courage, according to the stakeholders. Nevertheless, most stakeholders thought that crucial decisions are not being taken, for two reasons. Firstly, health care, and specifically integrated care, is not a high-profile public issue, which means politicians are not held accountable. Secondly, the required changes might contrast with the political preference of the decision-makers that are often contingent on ideological preference. For example, some interview participants questioned whether, the federal Minister of Health, from a liberal party at the time of the interviews (2019), would truly have deemed a mixed provider payment model a priority, as the current fee-for-service model corresponds to her liberal vision, while a system based more on capitation does not.

*“The problem for this Cabinet is that it’s a right-wing VLD Cabinet [liberal party] coming after a PS Cabinet [socialist party]. And for a VLD Cabinet to implement a reform that is decided by the PS Cabinet it’s not easy; […] And for them it’s not logical to force individual providers to work as a full consortium. It’s counter-natural as we say in French. It’s not natural for them to do that, to put forward a system of bundled payment, which is not payment for services, fee for services, which is really promoting enterprise, entrepreneurship. But this type of thing, bundled payment, is not so easy to understand by people. Everything is against a VLD logic, that’s why of course.” (IV26)*

A similar conclusion can be drawn from the developments in e-health, where the initial plan (set up under the reign of the former socialist minister) to develop a central common patient record accessible to all health care workers was cancelled in order to give private players on the market the chance to do so, which has slowed down progress.

### Relationships and linkages between macro-level factors

**Figure 2:**
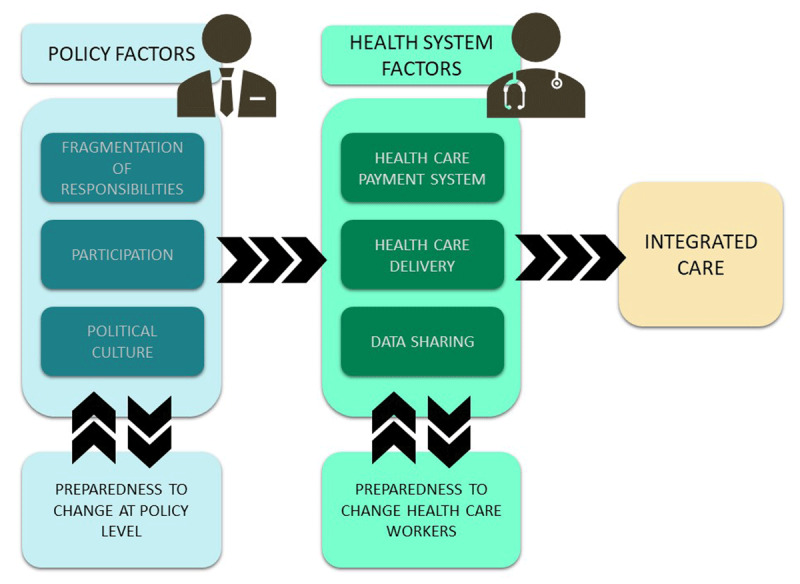
Relationships and linkages between macro-level factors.

The complex relationships and linkages between the macro-level factors are illustrated in ***Figure 2***. Integrated care forms the ultimate goal and is firstly influenced by factors in the health care system: health care delivery, data sharing and the health care payment system. These factors are in their turn influenced by policy factors: fragmentation of responsibilities, participation and political culture. To achieve integrated care, these factors need modification, and therefore a preparedness to change is essential at both the health care and policy levels. However, it is not only the policy factors that influence the health care system factors, as there are also interactions within the health and policy sector. The health care payment system, for example, influences health care delivery, due to the fee-for-service system which discourages task delegation. Similarly, the fragmentation of responsibilities influences political culture, as the division of political responsibilities means large-scale reforms are near impossible in this fragmented country in which negotiation is not just fostered, but also indispensable and in which the political structure almost solely allows gradual change in small steps.

## Discussion

Although multiple policy initiatives to scale-up integrated care in Belgium have been rolled out over the last decade, the stakeholders still evaluate the current level of implementation of integrated care to be low. This paper provided an analysis of the macro-level barriers to and facilitators of the scale-up of integrated care for chronic diseases in Belgium as perceived by the stakeholders and identified three main findings. Firstly, a dearth of joint priorities among stakeholders was reported, resulting in the lack of a clear aim at the health system-level. Secondly, there were multiple restraining factors at the health care organization level, such as the fee-for-service reimbursement system, with fragmentation and corporatism in budget allocation, and the slow development of integrated e-health solutions to data sharing. Lastly, the preparedness for change at the policy level is affected by the complex division of responsibilities between the federal and federated entities as the main barrier, because different aspects of the care continuum are governed by policies formulated at different levels of government, which often prove difficult to align. These three barriers in combination severely complicate the integration of care for chronic diseases care.

Our findings show that there is much interaction and interdependence between the different factors influencing integrated care, and this occurs both within and between the health care and policy levels. For instance, in order to enable health care workers to cooperate, data sharing is crucial. This can be stimulated or discouraged by the payment system. However, the fragmentation of responsibilities is important as well, as currently both the federal and the regional governments act on this topic. Finally, the political culture influences data sharing too, as one needs to define how such a shared patient record should look like. Consequently, to achieve the goal of integrated care, coordinated adaptations of all factors will be required. Initial steps have been taken to find solutions for these multiple interconnected issues, but these solutions will need to be complemented and coordinated, in order to be comprehensive.

Our study is in line with other analyses of the Belgium context, as well as with similar analyses in other settings. Previous research in the Belgian context has acknowledged the importance of a strong e-health system [32,33], adequate payment systems, such as all-inclusive payments or pay-for-coordination [33,34,35], and the need to involve all stakeholders [33]. Our study added to these the importance of assembling responsibilities at one government level, which becomes more prominent and urgent when policy initiatives at the different levels stumble. A similar conclusion was reached by a group of political scientists studying the pilot projects: “The way the multi-layer federal context in which the actions had to happen was designed created blockages, hindering the implementation process” [36] Research in other contexts points to the same generic elements, such as applying a comprehensive systems perspective [37], a shared vision [3,18], political leadership [18,37,38], evidence-based evaluation [3,37,38,39] and dedicated organizational capacity, in terms of both personnel and resources [18]. This study added to the international literature the importance of the link between the policy level and the health care level, as well as an emphasis on the interdependence of the different factors influencing the scale-up of integrated care.

In contrast to previous research, stakeholders in this study placed much of the accountability for the quality of care for people with chronic disease at the political level. Critics might say that, by doing this, health care providers deny their own responsibilities. And indeed, some professionals, particularly the doctors, fear change, because their income would probably be impacted [27]. However, multiple bottom-up projects have been rolled out [28,40] and continue to face the same macro-level barriers, which can only be overcome by new laws and regulations, as pointed out by various opinion makers, such as academics, the former head of the NIHDI and representatives of professionals [41,42,43]. The current concertation model of finding common ground to appease groups with different positions and search for compromises has proven to be ineffective, as it has resulted in the fragmentation of responsibilities, which is clearly at odds with the goal of integrated care. Political courage and commitment is needed to overcome disagreements and establish a strong and integrated health care system.

One strength of our study lies in the heterogeneity of the stakeholders interviewed, including high-level decision-makers. A broad range of stakeholders was interviewed: after actively seeking to include all stakeholders involved in decision-making on chronic care in Belgium, all key players took part, with no organization refusing to be involved. Moreover, an equilibrium was reached between high-level stakeholders on the one hand, with more political power, and technical staff on the other, who were able to explain the nuances of the barriers to and facilitators of integrated care. Another strength was our methodology, which considered three different policy initiatives in the interviews, allowing us to get insight in concrete experiences, identify concrete examples, but also to go beyond the policy-specific contextual factors. Both of these points of strength resulted in a broad vision of the barriers to and facilitators of integrated care in Belgium. This study had its limitations. One limitation concerns the stakeholder selection from Flanders and the federated level solely. Because of the difference in culture, language and context-specific barriers, Brussels, Wallonia and the German-speaking part of Belgium were out of scope of this study. When considering reforms the views and expectations of stakeholders in these regions should be explored and considered. A second and third limitation is that one stakeholder retracted their data after the interview, while a few high-level stakeholders were difficult to reach. These barriers were compensated for by asking the participants to share their perceptions of other stakeholders and their relations with them. However, this meant we should only interpret the data at the level of the group of stakeholders rather than at the individual level, which was how we applied it.

The analysis in this study has implications which could inform further endeavours to improve care integration in Belgium and beyond. Firstly, the financing system seems to potentially provide important leverage in directing change in health care delivery towards integrated care. There was significant evidence to suggest that policymakers should diversify the current fee-for-service model and move towards a mixed financing system that awards collaboration and chronic care quality. Secondly, as all of the factors influencing integrated care interact with each other, policymakers should apply a system-wide, comprehensive approach [36,44]. In this regard, the first step should be to locate the decision-making power on health care at one government level. The subsequent steps should consist of the development of a shared long-term vision on chronic care and implementing a change management strategy. Transparent decision-making and political courage will be needed, while support by the public will also be crucial. In this respect, the COVID-19 epidemic could have positive side effect, as it has made the importance of health care and integrated care in particular much more visible and has uncovered the deficiencies of the current system [45].

## Conclusion

The scale-up of integrated care is influenced by health care system factors such as the health care payment system, health care delivery and data sharing, as well as by policy factors such as the fragmentation of responsibilities, participation and political culture. All of these factors interact with each other, while the preparedness to change at both the policy level and health care level will be key to triggering a transformation leading to integrated care. Multiple policy initiatives have fostered integrated care for specific chronic diseases in Belgium and proposed or implemented adaptations to the system. However, important barriers, such as the financing system and the fragmentation of responsibilities, are hindering a much needed change to the entire Belgian health care system and a shift towards integrated care. Political commitment within this debate will be crucial in this regard.

## Appendix

**Appendix 1:** Code tree.

APPENDIX

Administration

Consultation

E-health

Data sharing

Fragmentation within e-health

Market vs central EHR offered by government

Mindset

Only for medical

Privacy

Financing

Budget

Costs for patients

Financing system

Capitation system

Fee for service

Mixed financing system

Pay for quality

Silo model

Supply vs demand-oriented system

Maisons médicales

Mentality to healthcare

Belief or unbelief in need for change

Change management

Different understanding integrated care

Self-interest

Participation & Collaboration

Equal partners

Fragmented field (meso-macro players)

Integration through care cotinuum

Knowing each other

LMN

Mentality to participate or collaborate

Role of the patient or citizen

Vertical integration

Voluntarism

Political system – reality

Competency split between governments

Governance

Decision process

Equity (vulnerable groups)

Interactive governance

Intersectoral approach

Multi-level governance

Transparency

Leadership

Local authorities

Policy process

Political preference

Political will (political support)

Time needed for research vs fast results wanted

Population health approach

Autonomy of HCW

Big data for population health approach

Kaiser permanente

Prevention

Primary care

Protocol care

Tailored interventions for patient

Task delegation

Training & education
